# 

**Published:** 1883-04

**Authors:** 


					﻿WL, III.
J^tir1883.
NO. 4.
hjhe
HOMEOPATHIC pH^iClAH,
A MONTHLY JOURNAL OF MEDICAL SCIENCE.
“ Magna est vcrilas, et prcEvqlebit."
EL CT. ZLIEOZElL jve. id.,
EDITOR.
CONTENTS:
(See inside of Cover.)
PHILADELPHIA:
No. 2109 CHESTNUT STREET.
2ST E "W YORK:
A. L CHATTERTON, PUBLISHING COMPANY, Publisher’s Agents.
LOKDON:
ALFRED HEATH & COf, Sole Agents for Great Britain.
114 Ebury Street, Eaton Square.
Yearly Subscription, $2.50, invariably in advance.
Entered at the Philadelphia Post-Office as Second-Class Mail Matter.
				

